# The Maudsley environmental risk score for psychosis

**DOI:** 10.1017/S0033291719002319

**Published:** 2020-10

**Authors:** Evangelos Vassos, Pak Sham, Matthew Kempton, Antonella Trotta, Simona A. Stilo, Charlotte Gayer-Anderson, Marta Di Forti, Cathryn M. Lewis, Robin M. Murray, Craig Morgan

**Affiliations:** 1 Social, Genetic & Developmental Psychiatry Centre, Institute of Psychiatry, Psychology & Neuroscience, King's College London, London, UK; 2 State Key Laboratory of Brain and Cognitive Sciences, Department of Psychiatry and Centre for Genomic Sciences, University of Hong Kong, Hong Kong, China; 3 Department of Psychosis Studies, Institute of Psychiatry, Psychology & Neuroscience, King's College London, London, UK; 4 Tony Hillis Unit, South London & Maudsley NHS Foundation Trust, London, UK; 5 Health Service and Population Research, Institute of Psychiatry, Psychology & Neuroscience, King's College London, London, UK; 6 Department of Psychiatry, Experimental Biomedicine and Clinical Neuroscience (BIONEC), University of Palermo, Palermo, Italy

**Keywords:** Environment, liability, psychosis, risk prediction, schizophrenia

## Abstract

**Background:**

Risk prediction algorithms have long been used in health research and practice (e.g. prediction of cardiovascular disease and diabetes). However, similar tools have not been developed for mental health. For example, for psychotic disorders, attempts to sum environmental risk are rare, unsystematic and dictated by available data. In light of this, we sought to develop a valid, easy to use measure of the aggregate environmental risk score (ERS) for psychotic disorders.

**Methods:**

We reviewed the literature to identify well-replicated and validated environmental risk factors for psychosis that combine a significant effect and large-enough prevalence. Pooled estimates of relative risks were taken from the largest available meta-analyses. We devised a method of scoring the level of exposure to each risk factor to estimate ERS. Relative risks were rounded as, due to the heterogeneity of the original studies, risk effects are imprecisely measured.

**Results:**

Six risk factors (ethnic minority status, urbanicity, high paternal age, obstetric complications, cannabis use and childhood adversity) were used to generate the ERS. A distribution for different levels of risk based on simulated data showed that most of the population would be at low/moderate risk with a small minority at increased environmental risk for psychosis.

**Conclusions:**

This is the first systematic approach to develop an aggregate measure of environmental risk for psychoses in asymptomatic individuals. This can be used as a continuous measure of liability to disease; mostly relevant to areas where the original studies took place. Its predictive ability will improve with the collection of additional, population-specific data.

## Introduction

Patient-tailored risk prediction is routinely applied in medicine and prediction models have been developed for a range of conditions like cardiovascular disease and diabetes (Wilson *et al*., 1998; Assmann *et al*., 2002; Wilson *et al*., 2007; Hippisley-Cox *et al*., 2008). These models use a combination of risk factors, including anthropometric traits (e.g. BMI, blood pressure), lifestyle (e.g. smoking), biochemistry tests (e.g. glucose or cholesterol levels) and family history of illness. These prediction models are included in clinical guidelines for prevention [e.g. cardiovascular disease: risk assessment and reduction, including lipid modification (CG181) or familial breast cancer (CG164), https://www.nice.org.uk/] and are increasingly advocated in public health (Damen *et al*., 2016).

Presymptomatic risk prediction is not common practice in psychiatry, despite extensive research in psychosis suggesting that early intervention can improve outcomes (Marshall *et al*., 2005; Fraguas *et al*., 2014) and delay or even prevent the onset of psychotic disorders (Stafford *et al*., 2013). Hence, any tool that can identify those at high risk of onset of psychosis or poor outcomes has potentially important public health and clinical applications.

Given the high heritability of schizophrenia (Sullivan *et al*., 2003; Lichtenstein *et al*., 2009), risk prediction algorithms to date have been typically based on genetic evidence (So *et al*., 2011) or single demographic factors, such as sex or ethnicity. With the advent of genome-wide association studies (GWAS), molecular data have been used to measure genetic predisposition directly. Associated polymorphisms individually have little predictive power, but the polygenic risk score (PRS), an aggregate measure of genetic loading combining thousands or tens of thousands of polymorphisms, has been more promising in risk prediction (Dudbridge, 2013). In the latest large meta-analysis of GWAS of schizophrenia, the PRS explained about 7% of the variance in the liability for schizophrenia in the general population (Schizophrenia Working Group of the Psychiatric Genomics Consortium, 2014), which is a more efficient predictor than single genetic risk factors (Vassos *et al*., 2017).

A number of environmental exposures have been identified that, individually, have been associated with an increased risk of psychosis. We envisage that an environmental risk score (ERS), as an estimate of the cumulative load of established environmental risk factors, would give a more accurate estimate of risk. This will facilitate research to improve our understanding of the overall impact of environmental factors and their interaction with genes in the development of psychosis; a necessary step before combining the total genetic and environmental information to improve risk prediction in asymptomatic individuals.

There is no consensus to date on the optimal way of estimating cumulative environmental risk for psychosis. Previous efforts to combine environmental risk factors have focused on predictive models for schizophrenia severity (Stepniak *et al*., 2014), cortical thickness (Neilson *et al*., 2017) or conversion to psychosis in individuals at familial high-risk for schizophrenia (Padmanabhan *et al*., 2017). These studies differed on the number and choice of the included environmental risk factors, their relative contribution, and the method of calculating the aggregate risk score; the choices largely depended on the data available in each study. Other existing risk calculators focus on the prediction of psychosis among individuals who have already developed a prodromal risk syndrome (Cannon *et al*., 2016; Carrion *et al*., 2016) or patients accessing secondary mental health care (Fusar-Poli *et al*., 2017).

To develop an ERS not limited by specific sample characteristics, independent of the onset of prodromal or other significant mental health symptoms, we sought to synthesise the available evidence and critically appraise conceptual and methodological issues in combining different environmental factors into a single risk score.

## Methods

### Selection of environmental factors

To select candidate environmental risk factors for psychosis to be included in the ERC, we modified the Venice criteria for the assessment of cumulative evidence on genetic associations (Ioannidis *et al*., 2008). For each factor, the robustness of the evidence for an association with risk of psychosis was determined by: (1) the amount of evidence (large-scale studies), (2) replication (extensive replication, with little inconsistency, and a well-conducted meta-analysis of all available data) and (3) steps to minimise bias in individual studies (e.g. due to selective reporting). To develop a practical and generalisable ERS, we added two additional criteria: (4) relatively easy to collect reliable information (based on a simple history from the patient or a family member) and (5) exposure preceding the onset of illness (to be relevant to a risk prediction model).

### Search strategy, data extraction and identification of effect size

Our search was performed according to the Preferred Reporting Items for Systematic Reviews and Meta-analyses (PRISMA) Statement (Moher *et al*., 2009). Potential studies were identified by a comprehensive search of the electronic databases PubMed, Embase and PsychINFO. Terms related to environmental risk in general or each putative risk factor (i.e. ethnic minority or migration or urban* or paternal age or pregnancy complication or obstetric complications or perinatal infection or child* adversity or child* trauma or child* abuse or child* victimization or cannabis or substance use or drug abuse or stressful life events or recent life events) were combined with the terms psychosis or psychotic disorders or schizophrenia or schizo*. The search was initially limited to systematic reviews or meta-analyses of studies of putative risk factors to select the most recent large meta-analysis.

To evaluate if the meta-analyses provide a good summary of all the available evidence, we repeated the above search from the publication year of each selected meta-analysis to the present, without restricting the article type, and we examined relevant titles/abstracts as well as reference lists from the recently published reviews. Effect sizes from the new studies were compared with those from the meta-analyses to see whether new evidence corroborated estimates to be used in our risk model.

### Construction of environmental risk score

We developed an easy to use method to pool the existing evidence together to construct an ERS, which can serve different purposes (i.e. a quick estimate of an individual's risk for use in a clinical setting or a quantitative measure of the aggregate environmental risk for research purposes). This method involves generating a weighted sum of environmental exposures present, similar to the Framingham Risk Score (D'Agostino *et al*., 2008), based on effect sizes taken from the corresponding meta-analyses. These are presented as odds ratios (OR), incidence rate ratios (IRR) or relative risks (RR), depending on the study design. As psychosis is a rare outcome, they were all considered a good approximation of RR. Crude effect sizes, when available or minimum adjustment (e.g. for age and sex) were used from the individual studies, to improve comparability between studies. The ERS was constructed according to the following steps:

(1) As relative risks (RR) in the meta-analyses are expressed comparing exposed (risk factor as binary or ordinal variable) and non-exposed individuals, and as only a minority of the population would not be exposed to any risk, we calculated RR relative to the ‘average individual’ of the general population (RR_scaled_). Hence, the weighted mean of RR_scaled_ is 1, while individuals in the low exposure group would have an RR_scaled_ < 1 (below average risk of disorder). More explicitly, for each environmental factor, we used the meta-analyses to estimate the proportion (*p*_*j*_) of the population within each exposure group (*j*) and then we scaled the RR according to the formula RR_scaled_ = RR_*j*_/∑(RR_*j*_ × *p*_*j*_). In the case of urbanicity and cannabis, risk has been expressed as a continuous function of the exposure. However, as this level of information is not easily available, we split exposure for simplicity to three levels (none/low, medium, high exposure) and estimated log(RR) from the corresponding beta coefficients (detailed methods in online Supplementary material).

(2) To construct a simple scale avoiding fractional numbers and taking into account the fact that effect sizes based on meta-analyses are approximations due to heterogeneity, measurement error and context contingency, we multiplied log(RR_scaled_) by a constant of 10 and then we rounded to the nearest half-integer.

(3) The combined ERS is simply the sum of the individual points in this scale, replacing missing values with 0. An approximation of the RR of an individual compared to the ‘average’ person of the general population can be derived by dividing the ERS by 10 and estimating its antilogarithm to base 10 (RR ≈ 10^ERS/10^).

As effect sizes for our model were taken from separate meta-analyses, the ERS we calculated was based on the assumption that the risk factors are uncorrelated. To test for the effect of potential correlation between risk factors, we simulated data under three different scenarios: (1) each factor independent of the others, (2) people in the high urbanicity groups having 10% increased chances of being exposed to each of the others risk factors and (3) as above with 20% increase of all the risk factors in the high urbanicity group. We performed logistic regression of case-control status under each scenario, which allowed effect size estimation for each factor adjusted for the others. We also estimated each model's significance and variance explained (online Supplementary material).

## Results

We identified six environmental risk factors fulfilling our inclusion criteria: minority ethnic group, urbanicity, high paternal age, obstetric complications, cannabis use and childhood adversity (Table 1). We also identified meta-analyses on traumatic brain injury (OR = 1.65) (Molloy *et al*., 2011), *Toxoplasma gondii* infection (OR = 1.81) (Sutterland *et al*., 2015), cigarette smoking (OR = 3.22) (Gurillo *et al*., 2015). These were not included in the ERS either due to insufficient evidence, difficulty to establish exposure in a clinical interview (e.g. IgG antibodies for *T. gondii*) or potential of overlap with other included risk factors (e.g. smoking and substance abuse). Stressful events related to education, work, reproduction, housing, finances, crime, health, relationships and death have been implicated in the onset of psychosis. A meta-analysis of 13 studies (Beards *et al*., 2013) estimated an overall weighted OR of life events in the period prior to psychosis onset of 3.19. However, as the authors note, the sample size and methodological quality of the majority of studies were low, which urges caution in interpreting the results. In addition, as recent life events are time-dependent, they cannot be incorporated in the same model as other risk factors; however, they have a role in identifying periods of high risk of the first episode of psychosis. Each factor included in the ERS model is presented separately below.

**Table 1.** Meta-analyses of environmental risk factors

Risk factor	Study	*N* cases	Sub-categories	RR	95% CI
Migration	Bourque *et al*. (2011)	38 716	First generation^a^	2.3	2.0–2.7
			First Black	4	3.4–4.6
			First other	2	1.6–2.5
			First White	1.8	1.6–2.1
		28 449	Second generation	2.1	1.8–2.5
Urbanicity	Vassos *et al*. (2012)	47 087		2.39^b^	1.62–3.51
Paternal age	Miller *et al*. (2011)	16 204	<25	1.06	1.01–1.11
			25–29	1	NA
			30–34	1.06	1.01–1.1
			35–39	1.13	1.08–1.19
			40–45	1.22	1.14–1.3
			45–50	1.21	1.09–1.34
			>50	1.66	1.46–1.89
Obstetric complications	Cannon *et al*. (2002)	1294	Birth weight <2.500 g	1.67	1.22–2.29
Cannabis	Marconi *et al*. (2016)	4036		3.9^b^	2.84–5.34
Childhood adversity	Varese *et al*. (2012)	5698		2.78	2.34–3.31

a Corresponds to any ethnic minority. This includes the three subcategories (Black, White, other) below. It can be used instead of specific values if the ethnic background is unknown.

b RR of highest *v.* lowest exposure, assuming a linear increase of the risk.

### Minority ethnic group

An association between migration and schizophrenia has been replicated in many countries and the evidence indicates that risk is elevated in some (but not all) minority ethnic groups (i.e. settled migrants and subsequent generations born in the new countries) (Cantor-Graae and Selten, 2005), including consistent reports of high incidence rates among black populations in the UK (Fearon *et al*., 2006). The largest meta-analysis of incidence studies on migration/minority ethnic groups, providing information on 38 716 cases (Bourque *et al*., 2011), yielded mean-weighted age- and sex-adjusted IRRs of 2.3 (95% CI 2.0–2.7) and 2.1 (95% CI 1.8–2.5) for first- and second-generation migrants respectively. More specific IRRs were estimated for subgroups (e.g. IRR of 4 for Black, 1.8 for White and 2.0 for other first-generation immigrants). Similar IRRs were estimated in a recent meta-analysis of incidence studies (Selten *et al*., 2019), which also gives estimates depending on the place of origin and place of residence.

### Urbanicity

The association between population density and risk of psychosis, especially schizophrenia, is well established, at least in northern European cities. Despite the different methodologies used for the measurement of urban exposure, several studies have confirmed that living in densely populated, urban environments is associated with increased the risk of schizophrenia or psychosis in general. In a previous meta-analysis of population register studies comprising a total of 47 087 cases with psychosis (Vassos *et al*., 2012), we calculated the pooled OR for psychosis comparing the most urban with the most rural environment to be 2.39 (95% CI, 1.62–3.51). Based on the United Nations World Urbanization Prospects report of an almost equal distribution between urban and rural environments in the global population (United Nations. Department of International Economic and Social Affairs., 2014), we split the distribution into three equal tertiles and estimated the mean OR for each (detailed methods in online Supplementary material).

### Paternal age

Advanced paternal age has been repeatedly associated with increased risk of schizophrenia and non-affective psychosis (Malaspina *et al*., 2001). The latest meta-analysis (Miller *et al*., 2011) pooled crude estimates from 12 studies including 23 301 cases with schizophrenia. As the effect sizes (RR, OR) in cohort and case-control studies were very similar, we used the estimates from the combined studies to use the maximum amount of data. The observed increase in the risk was not linear as the authors, comparing risk in 5-year intervals, found a sharp increase in fathers over 50 year old. As some age groups had very similar risk estimates, we collapsed the data in three groups: below 40 (baseline), 40–50 and over 50.

### Obstetric complications

Obstetric complications (OCs), which include a wide range of events such as complications of pregnancy, abnormal foetal growth and complications of delivery, are associated with about two-fold increased risk of schizophrenia. In the largest meta-analysis of eight prospective population-based studies comprising 1923 cases with schizophrenia (Cannon *et al*., 2002), ORs for the presence *v.* absence of 30 different complications as dichotomous variables are given (range 0.63–7.76). Due to the difficulty of collecting reliable information on most OCs in a clinical interview, we chose birth weight below 2.5 kg (OR = 1.67) as a relatively easy to remember proxy of OCs, based on the effect size and the proportion of the population exposed to the risk. Similar ORs were estimated in a larger study of incident cases based on the national registers in Sweden and Denmark (Abel *et al*., 2010).

### Cannabis use

Current evidence shows that high levels of cannabis use are associated with an increased risk of psychosis; indeed, our meta-analysis including 4036 individuals with psychotic diagnoses or symptoms confirmed evidence of a dose–response relationship between the level of use and the risk for psychosis (Marconi *et al*., 2016). The estimated pooled crude OR for the risk of psychoses among the heaviest cannabis users compared with non-users was 3.9 (95% CI 2.84–5.34). If quantitative information on cannabis exposure is available, the expression of the association in a linear equation (similar to urbanicity) allows estimation of the risk for psychosis at different exposure levels. We estimated OR for the unexposed, assuming they were 70% of the population (European Monitoring Centre for Drugs and Drug Addiction, 2017) and we split the exposed individuals to two equal groups, representing 15% each (detailed methods in online Supplementary material).

### Childhood adversity

One widely replicated set of environmental risk factors for psychosis is exposure to adverse experiences in childhood, such as physical or sexual abuse, or parental separation (Trotta *et al*., 2015). In the most comprehensive meta-analysis of 36 studies (Varese *et al*., 2012), including 5698 psychotic patients, any adversity was associated with an increased risk of psychosis, with an overall OR of 2.78 (95% CI = 2.34–3.31). The magnitude of the effect was comparable across different study designs including case-control, population-based cross-sectional, and prospective studies and those that used retrospective and prospective measures of adversity (OR = 2.72, 2.99 and 2.75 respectively), hence for the ERS we used the overall OR of 2.78.

### Proposed Maudsley environment risk score for psychotic disorders

Taking the evidence collected, we constructed an ERS by summing the rounded log risk ratios. The ERS can take a value between −4.5 (lowest risk) and 16 (maximum risk). The numeric values for estimating ERS according to the level of exposure to each risk factor are presented in Table 2. The ERS can be used as a continuous variable of aggregate environmental risk or can be applied to any individual to estimate premorbid relative risk for psychosis.

**Table 2.** Values for estimation of Environmental Risk Score

Risk factor	Sub-categories	RR from M-A	Proportion of population (%)	Scaled log(RR)	ERS
Ethnic minority	Native	1	92.4	−0.04	−0.5
	Black	4	1.3	0.56	5.5
	White	1.8	2.8	0.22	2
	Other	2	3.5	0.26	2.5
Urbanicity (place of birth)	Low	1.16	33.3	−0.14	−1.5
	Medium	1.55	33.3	−0.01	0
	High	2.07	33.3	0.11	1
Paternal age	<40	1	92.1	−0.01	0
	40–50	1.17	7.1	0.06	0.5
	>50	1.60	0.8	0.19	2
Obstetric complications	Birth weight ⩾2.5 kg	1	96.4	−0.01	0
	Birth weight <2.5 kg	1.67	3.6	0.21	2
Cannabis	No exposure	1	70	−0.12	−1
	Little/moderate	1.41	15	0.02	0
	High exposure	2.77	15	0.32	3
Childhood adversity	No exposure	1	73	−0.17	−1.5
	Any exposure	2.78	27	0.27	2.5

For example, if a person is a white migrant (2 points), born in an urban environment (1), to a 37-year-old father (0), with no obstetric complications (0), moderate cannabis use (0) and unknown childhood adversity (0), the ERS would be 3, which corresponds to a relative risk (RR) of 2 compared with the general population. Similarly, in a scenario of an individual coming from the dominant ethnic group (−0.5 points), born in a rural area (−1.5), to a father over 50 years (2), with low birth weight (2), no history of smoking cannabis (−1) and prior exposure to childhood adversity (2.5), the ERS would be 3.5, corresponding to an RR of 2.24.

To visualise the range and distribution of ERS, we performed 1 million permutations, randomly allocating exposure to the different levels of environmental risk, according to the proportion of the population that belonged to each group in the original meta-analyses. The distribution is skewed with the majority of individuals belonging to the low or moderate risk groups and only a few individuals being high risk (e.g. only 2% of the population has an RR of 4 or more). Analysis of simulated data under different levels of correlation between risk factors and urbanicity (0, 10 and 20%) showed minimal change in the predictive power of ERS (online Supplementary material).

## Discussion

This is the first effort to develop an environmental risk score for psychosis which can be applied to any individual before the onset of any symptoms, based on data from a systematic search of the literature, rather than on data available in a single sample. Unlike previous approaches of counting risk factors, which are then assumed to contribute an equal amount of risk, the Maudsley ERS weights each factor by the best estimate of its effect size. This is a powerful approach, making use of most of the available evidence to estimate premorbid risk for psychosis. The ERS, in combination with family history or molecular genetic data, when available, has the potential to improve risk prediction.

The proposed ERS can be utilised in research by giving the best available estimate of the aggregate environmental risk for psychosis, which could explain an estimated 7% of the variability in liability to disease (Gillett *et al*., 2018). Before considering its potential to improve risk prediction in clinical practice, similar to the Framingham risk score for cardiovascular disease or diabetes (Wilson *et al*., 1998; Wilson *et al*., 2007), it needs to be validated in clinical samples. We identified a number of limitations in current evidence that needs to be addressed.

Although we used effect size estimates from the latest meta-analysis for each risk factor, evidence is constantly accumulating with new research findings being published. With our search, we identified new studies on ethnic minorities, paternal age and childhood adversities, published since the included meta-analyses. With few exceptions, confidence intervals of these studies largely overlap with the pooled effect sizes; hence they would not substantially alter the estimates. Nonetheless, we identified a need for an update of the meta-analyses and subsequently the risk score will need modification; therefore, we should consider the current ERS as the first version of an indicator of risk that will need to be regularly updated based on new research findings.

A second issue is the generalisability of the findings, given most of the published research on psychosis is based in northern Europe, America and Australasia. For example, we know that urban birth is associated with psychosis risk in northern Europe, but we cannot be sure that the same applies to India or Africa or the Americas. Similarly, we have estimates of increased risk of psychosis for black minority ethnic groups in London, but we do not have adequate data for ethnic minorities in Southern Europe (DeVylder *et al*., 2018). Hence, to have a more global view of risk factors it is essential to perform studies estimating psychosis risk in different parts of the world. At present, we expect that the predictive validity of the model will be higher in countries where the original studies were conducted; mainly Northern Europe. When local data is available (e.g. estimates of urbanicity or cannabis risk for psychosis in a specific area), there is the possibility of replacing the RR from the summary data presented in this paper with local estimates. For example, the latest meta-analysis on ethnic minority status (Selten *et al*., 2019), reports RR depending on the place of residence, which can be used to tailor the risk score to specific areas.

Unlike in GWAS, where risk for each variant has been measured in the same dataset, estimates for the effects of each environmental factor are taken from different studies; and the measures of environment are more heterogeneous. We noticed that individual meta-analyses pool together studies with very variable effect sizes, which reflects either a degree of misestimation of the true effect, or population-specific differences. Consequently, estimated effects are more crude and confidence intervals often wide. Rounding the ERS to the closest half-integer is a reflection of this uncertainty of the pooled effect sizes.

The statistical model to combine risk factors in an aggregate score is based on the assumption that risk factors are independent. However, environmental factors are often correlated with each other (Guloksuz *et al*., 2018). For example, ethnic minority groups more often live in cities and individuals with older parents may have more frequent obstetric complications. In this case, some of the risks for psychosis is double-counted. Since the effect sizes have been taken from separate meta-analyses and individual studies adjusted for different factors according to the data available in each cohort, it is not simple to account for these intercorrelations. To test the effect of correlated risk factors to the model, we performed simulations under three different scenarios of correlations with high urbanicity and interestingly we did not find large differences. However, we acknowledge that intercorrelation of risk is an important limitation in this effort to produce an environmental risk score and we endorse proposals for exposure-wide systematic approaches in future studies design (Guloksuz *et al*., 2018).

**Fig. 1.**
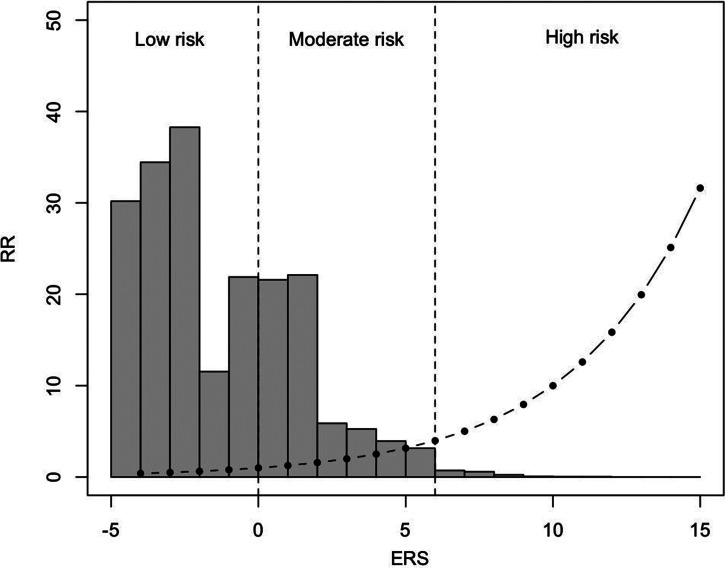
Distribution of ERS and corresponding RR in the general population. The dots represent the ERS and the corresponding relative risk for psychosis and the grey bars a histogram of the distribution of the population at different levels of risk based on 1 million permutations assuming that the risk factors are independent. Approximately 62% of the total population is at low risk (RR ⩽ 1), 34% at moderate risk and only 4% are at high risk (here defined as RR ⩾ 4).

One issue that we tried to address is the optimal method for combining different risk factors. In this paper, we added risk ratios on the log scale, similar to the approach taken for the Framingham Risk Score (Wilson *et al*., 1998; D'Agostino *et al*., 2008) and the method used for the PRS (Dudbridge, 2013). There is a possibility that some risk factors interact with each other or that there is a ‘ceiling’ on their effect when many risk factors are present, but in the absence of concrete evidence, interactions were not included in the model. Also, only a negligible proportion of the population will have a combination of too many risk factors (right tail of distribution in Fig. 1) to test for a ceiling effect; hence we advise caution in the interpretation of high-risk estimates.

Family history (although easy to collect and an important risk factor) was not included in the model for two reasons: (1) the risk related to family history can be conceptually divided to a genetic and a shared environmental component. The genetic risk is not relevant to this score and, given the availability of molecular genetic data and the increasing predictive power of PRS, there is an argument for keeping family history separate and include it for risk prediction as an alternative to GWAS data, when the latter is not available. (2) The environmental component of family history may largely overlap with the included risk factors (i.e. members of the same family to a large extent share risk related to urbanicity, ethnic minority status, exposure to cannabis, childhood adversity etc.); increasing further the problem of intercorrelations discussed above.

At this stage, we do not propose that the ERS can give an exact estimate of absolute risk for psychosis, but it can be useful in differentiating individuals in groups of low, moderate or high risk (Fig. 1). To apply the ERS for individual risk prediction, we need to make assumptions that risk is stable over time and similar in men and women because there is not enough data yet to estimate more precise risk by age groups or gender. To translate this to an estimate of the absolute risk for psychosis, more relevant to clinical practice, age of psychosis onset curves for men and women can be used. One benefit of our method is that we estimate RRs compared to the average person in the general population, not the ones with no risk. Hence, a person with few risk factors would be considered ‘protected’ against psychosis and would have an RR less than 1. This gives more realistic RR estimates and allows the inclusion of missing data in the model.

In summary, measuring the cumulative environmental risk is of importance given its potential to inform efforts to prevent the onset or persistence of psychotic disorders. We acknowledge that there are currently several limitations in the clinical utility for the proposed Maudsley Environmental Risk Score. The priorities we identified are the need to improve relative risk estimates by conducting studies that measure the effect of all the risk factors together to account for inter-correlations, to expand research outside Europe/North America, and to measure whether the risk from environmental exposures acts synergistically or interacts with genetic risk (measured through PRS or simply from family history). However, as the PRS has substantially improved prediction in comparison with single genetic factors and its clinical potentials start to become apparent (Vassos *et al*., 2017), we envisage that an environmental analogue will be equally valuable for research and eventually clinical purposes.

## Supplementary material

For supplementary material accompanying this paper visit https://doi.org/10.1017/S0033291719002319. click here to view supplementary material
