# Supplementary material for: The Maudsley environmental risk score for psychosis
Source: Psychol Med. 2019 Sep 19;50(13):2213–20. doi: 10.1017/S0033291719002319 (PMC7557157; doi:10.1017/S0033291719002319)
Supplement: Supplementary file 1 [file S0033291719002319sup001.docx]

**Supplementary Material**

**The Maudsley Environmental Risk Score for Psychosis**

Evangelos Vassos MD, Pak Sham MD, Matthew Kempton PhD, Antonella Trotta PhD, Simona A. Stilo PhD, Charlotte Gayer-Anderson PhD, Marta Di Forti MD, Cathryn M Lewis PhD, Robin M Murray FRS, Craig Morgan PhD

Correspondence: Dr Evangelos Vassos, SGDP Centre, IoPPN, King's College London, De Crespigny Park, London, SE5 8AF, United Kingdom, Email: Evangelos.Vassos@kcl.ac.uk

**Supplementary methods**

Estimation of relative risk (RR) for urbanicity in 3 levels of exposure

The model is based on our previous meta-analysis (Vassos et al. 2012), where we estimated a formula of risk as a continuous function of population density at birth, with the odds ratio of the most urban to the most rural individual estimated to be 2.39. If we know the rank (here denoted as u) of an individual’s population density at birth in a scale of 0 to 1, we can estimate the odds ratio from the formula log(OR) = log(2.39)*u. As we realise that it is difficult to know the exact rank of an individual’s urbanicity, to simplify the model, we divide the total population in 3 equal groups, each corresponding to 1/3 of the population, and we estimate the OR of the median individual in each of the groups (i.e. the u in the above formula takes the values 0.166, 0.5, 0.833 - which are respectively the median of the groups 0-0.33, 0.33-0.67, and 0.67-1). Then we applied the method of scale to the average individual as in the other risk factors.

Estimation of relative risk (RR) for cannabis in 3 levels of exposure

The model is based on our previous meta-analysis of studies of cannabis exposure and risk of psychosis (Marconi et al. 2016). Using the same method as urbanicity, we estimated risk as a continuous function of the degree of cannabis use, with the relative risk of the heavier cannabis user estimated to be 3.9 compared to the non-user. The difference between this and the previous model is that, although in urbanicity we assumed that the whole population lies in a uniform distribution of exposure, in cannabis we fitted 70% of the population to no (or minimal) use and allowed the remaining 30% to vary in a uniform distribution. This was split into two equal parts (representing 15% of the total sample each) and we fit log(OR) = 0 for the non-users, and log(OR) = log(3.9)*u with u taking the values of 0.25 and 0.75 for the little to moderate and the high exposure group.

Simulations to estimate RR if risk factors are correlated

To test the effect of correlation in our risk ratios, we simulated data under 3 different scenarios: 1) running 10 million permutations, we created a sample of cases and controls that fits the 1% prevalence of the disorder and the distributions of each risk factor according to the estimated proportion in the population as in table 1, with each factor independent of all the others. 2) We repeated the same procedure fitting the distribution of risk factors such that people in the high urbanicity groups had 10% increased chances of being exposed to each of the others risk factors (belonging to an ethnic minority, having an older father, obstetric complications, being a heavy cannabis user, having history of childhood trauma). The risks in the low urbanicity subsample were scaled down to achieve the expected overall prevalence of 1%. 3) We performed the above procedure fitting 20% increase of all the risk factors in the high urbanicity group. Then we performed a series of logistic regressions of case-control status with each of the factors separately and in single models under each of the 3 scenarios. This allowed effect size estimation for each factor adjusted for the others. We also estimated each model’s significance and variance explained (Nagelkerke R^2^).

**Supplementary Results**

In the first scenario (uncorrelated risk factors) the model with all the factors together gave similar RR estimates as the given RR from the meta-analyses we used for the ERS. The model with all risk factors separately explained more variance (5.5%) than the model with the risk summed in an ERS (4.6%). In the two scenarios of risk correlated with urbanicity we notice a drop (more pronounced in the 3^rd^ scenario) in the RR of the medium and high urbanicity groups with little difference in the other risk factors. However, the variance explained by ERS dropped only by a small amount from 4.6 to 4.5% (supplementary table 1).

**Supplementary Table 1.** Relative Risks of schizophrenia of the environmental risk factors under the 3 different scenarios.

| **Risk factor** | **Sub-categories** | **RR vs baseline** | | **RR separate^1^** | **RR uncorrelated^2^** | **RR 10% correlated^3^** | **RR 20% correlated^4^** |
| --- | --- | --- | --- | --- | --- | --- | --- |
| Ethnic minority | Native | 1 | | 1 | 1 | 1 | 1 |
|  | Black | 4 | | 4 | 4.02 | 3.94 | 4 |
|  | White | 1.80 | | 1.82 | 1.83 | 1.78 | 1.80 |
|  | Other | 2 | | 2 | 2 | 2 | 2 |
| Urbanicity (birth) | Low | 1 | | 1 | 1 | 1 | 1 |
|  | Medium | 1.34 | | 1.34 | 1.34 | 1.26 | 1.17 |
|  | High | 1.79 | | 1.80 | 1.80 | 1.58 | 1.39 |
| Paternal age | <40 | 1 | | 1 | 1 | 1 | 1 |
|  | 40-50 | 1.17 | | 1.16 | 1.16 | 1.16 | 1.16 |
|  | >50 | 1.60 | | 1.58 | 1.58 | 1.60 | 1.55 |
| Obstetric complications | No complications | 1 | | 1 | 1 | 1 | 1 |
|  | Birth weight <2.500g | 1.67 | | 1.68 | 1.66 | 1.65 | 1.64 |
| Cannabis | No exposure | 1 | | 1 | 1 | 1 | 1 |
|  | Little to moderate | 1.41 | | 1.44 | 1.44 | 1.41 | 1.42 |
|  | High exposure | 2.78 | | 2.79 | 2.79 | 2.78 | 2.80 |
| Childhood Adversity | No exposure | 1 | | 1 | 1 | 1 | 1 |
|  | Any exposure | 2.78 | | 2.76 | 2.76 | 2.76 | 2.75 |
|  |  |  | **Nagelkerke** **R2** | |  |  |  |
|  |  |  | All risk^5^ | | 0.055 | 0.055 | 0.055 |
|  |  |  | Risk Score^6^ | | 0.046 | 0.046 | 0.045 |
|  |  |  | |  |  |  |  |

^1^ RR when each risk factors was entered alone in a logistic regression model

^2^ RR in a model with all 6 uncorrelated risk factors predicting case-control status

^3^ RR in a model where high urbanicity increases by 10% the chance of belonging to the high-risk groups in all the other risk factors

^4^ RR in a model where high urbanicity increases by 20% the chance of belonging to the high-risk groups in all the other risk factors

^5^ Nagelkerke R^2^ case-control variance explained from a logistic regression model with all the risk factors separately

^6^ Nagelkerke R^2^ case-control variance explained from a logistic regression model with ERS as predictor
